# Two Single-Nucleotide Polymorphisms in *ADAM12* Gene Are Associated with Early and Late Radiographic Knee Osteoarthritis in Estonian Population

**DOI:** 10.1155/2013/878126

**Published:** 2013-03-28

**Authors:** Irina Kerna, Kalle Kisand, Ann E. Tamm, Jaanika Kumm, Agu O. Tamm

**Affiliations:** ^1^Department of Internal Medicine, University of Tartu, 51014 Tartu, Estonia; ^2^Department of Immunology, University of Tartu, 51014 Tartu, Estonia; ^3^Department of Sports Medicine and Rehabilitation, University of Tartu, 51014 Tartu, Estonia; ^4^Department of Radiology, University of Tartu, 51014 Tartu, Estonia

## Abstract

*Objectives.* To investigate associations of selected single-nucleotide polymorphisms (SNPs) in *ADAM12* gene with radiographic knee osteoarthritis (rKOA) in Estonian population. *Methods.* The rs3740199, rs1871054, rs1278279, and rs1044122 SNPs in *ADAM12* gene were genotyped in 438 subjects (303 women) from population-based cohort, aged 32 to 57 (mean 45.4). The rKOA features were evaluated in the tibiofemoral joint (TFJ) and patellofemoral joint. *Results.* The early rKOA was found in 51.4% of investigated subjects (72% women) and 12.3% of participants (63% women) had advanced stage of diseases. The A allele of synonymous SNP rs1044122 was associated with early rKOA in TFJ, predominantly with the presence of osteophytes in females (OR 1.57; 95% CI 1.08–2.29, *P* = 0.018). The C allele of intron polymorphism rs1871054 carried risk for advanced rKOA, mostly to osteophyte formation in TFJ in males (OR 3.03; 95% CI 1.11–7.53, *P* = 0.018). Also the CCAA haplotype of *ADAM12* was associated with osteophytosis, again mostly in TFJ in males (*P* = 0.014). For rs3740199 and rs1278279, no statistically significant associations were observed. *Conclusion.* *ADAM12* gene variants are related to rKOA risk during the early and late stages of diseases. The genetic risk seems to be predominantly associated with the appearance of osteophytes—a marker of bone remodelling and neochondrogenesis.

## 1. Introduction

Osteoarthritis (OA) is the most common joint disorder and represents a leading musculoskeletal health and socioeconomic burden [1]. The knee is one of the most affected sites [2]. Among recognized OA risk factors like age and overweight, the genetic background, as demonstrated in twins, is expected to play the significant role [3]. To date, several genomewide linkage analyses (GWAS) and numerous association studies of candidate genes have been performed to disclose genetic pattern of OA [4]. Despite promising evidence, only few genes like GDF5 and SMAD3 demonstrated proven susceptibility to OA [5] and these genes, in turn, interpret only a small part of the genetic contribution to the disease. 

Until now, promising but contradictory data are published for the association of a member of disintegrin and metalloproteinase family—*ADAM12* with the pathogenesis of OA [6, 7]. ADAM12 is an active proteinase, which is highly expressed in remodelling and fast-growing tissues such as the placenta and malignant tumours [8]. One of the splice variant of *ADAM12* was found to be overexpressed in human OA cartilage [9], and recently, we described the elevation of ADAM12 protein in serum of OA patients [10]. Additionally, one of the genetic variants—intronic single-nucleotide polymorphism (SNP) rs1871054 in *ADAM12* gene—was recognized as a risk predictor in multiplicative knee OA (KOA) model [11].

The aim of this study was to investigate four polymorphisms within *ADAM12* gene to assess the impact of previously reported findings on the risk of radiographic KOA (rKOA) in the Estonian cohort.

## 2. Patients and Methods

### 2.1. Subjects

A population-based sample was selected from three Family Doctor (FD) registers of the small towns of Elva and Võru, southern Estonia. The first questionnaire was mailed to all 1793 individuals, aged 35–57, registered with these FDs. Altogether 964 individuals responded (54%). Out of them 506 affirmed the presence of KOA problems, and the rest 459 disaffirmed it. Patients with diagnosed inflammatory arthropathies were not included in the research. Furthers 475 (among them 67% female) out of the 964 responders agreed to pass an in-depth clinical examination including inquiry, performance tests, X-ray radiography of both knee joints, and DNA collection. Thus we were able to assess radiographic status in 26.5% of the subjects of the selected age group.

Standardized weight-bearing anteroposterior radiographs of tibiofemoral joint (TFJ) and axial radiographs of the knee joint with patellofemoral (PFJ) in 60° flexion were used for radiographic assessment of knee OA. The two main features of rKOA, the presence of joint space narrowing (JSN) and osteophyte development, were estimated in both TFJ and PFJ according to the grading system (grades 0–3) of Nagaosa et al. [12]. The highest grade of JSN or OPH was regarded as the stage of OA in the corresponding joint. The highest grade of OA in TFJ or PFJ was regarded as the global stage of rKOA, and the highest grade of osteophytes in TFJ or PFJ was regarded as the global grade of osteophytes. Blood samples were collected for the assessment of bone and cartilage turnover markers and subsequent genetic investigation. In 33 subjects (14 women) blood DNA sample was not available. In four subjects (3 women) data of knee joints radiographic examination was missing. The final study group consists of 438 subjects (303 women), mean age 45.4 years. 

The study was approved by the Ethics Committee for Human research of the University of Tartu, and informed consent was obtained from all subjects.

### 2.2. SNP Genotyping

DNA was extracted from whole EDTA-blood by phenol-chloroform extraction [13]. TaqMan SNP Genotyping Assays (C_1419869_1, C_12049599_10, C_3077142_1, C_3077192_10, and C_1839361_1, Applied Biosystems, Foster City, CA, USA) were used to detect four SNPs in *ADAM12* (rs3740199, rs1871054, rs1278279, and rs1044122). Each sample was normalized to a concentration of 10 ng/*μ*L using DNAse-free water. The quantitative PCR assay was performed using an ABI PRISM 7000 SDS analyser with allelic discrimination software. Each assay well contained a 5 *μ*L reaction volume, consisting of 2.5 *μ*L of 20X TaqMan Universal PCR master mix (Applied Biosystems, Foster City, CA, USA), 0.06 *μ*L of 40x TaqMan primers, 1.5 *μ*L of DNAse-free water, and 1 *μ*L of DNA. The amplification was performed according to the standard protocol [14].

### 2.3. Statistical Analysis

The association between the SNPs and different radiographic features of KOA (global grade of OA, presence of OPH and JSN in TFJ and/or PFJ) was evaluated using the logistic regression model. The age, body mass index and sex of the subjects were used as the covariates in all statistical calculations. To correct for the effects of multiple comparisons, we used false discovery rate control by Benjamini and Yekutieli (B-Y method), which provides an acceptable balance of type I and II errors. Additionally the B-Y method provides intermediate critical values as demonstrated not only in simulations but also as applied to empirical genetic data analyses [15]. In the paper corrected *P* values are marked by asterisk.

 For estimation of associations between haplotypes of selected SNPs and OA radiological traits, we used the haplo.stats (haplo.cc) package. The Hardy-Weinberg equilibrium (HWE) was evaluated using the genetics package (HWE.test). All calculations were performed in the R environment ver. 2.4.0 (The R Foundation for Statistical Computing, Boston, MA, USA). The *P* value of 0.05 was designated as statistically significant. 

The allele frequencies of genotyped SNP were compared to those reported previously for the European population within the HapMap project [16] and the Nottingham cohort [17] using the Yates Chi-square test. 

Power estimates were performed using the Quanto ver. 1.2.4 software [18] with the following options: population risk of 12%, significance level 0.05, and log-additive inheritance model.

The results of linkage disequilibrium (LD) mapping were generated using Haploview software.

## 3. Results

### 3.1. Distribution of Radiographic Knee OA in Investigated Subjects

The prevalence of radiographic knee OA (grade 1–3) in investigated subjects was 63.6% (61.3% in males and 66.0% in females). 

The early features of rKOA in TFJ and/or PFJ were found totally in 225 out of 438 (51.3%, mean age 46.6) subjects and advanced stage of rKOA in 54 participants (13%, mean age 47.9) in investigated population. No difference in rKOA distribution was observed between two genders.

The summary of radiological evaluation of rKOA in both joints—TFJ and PFJ—is presented in Table 1. The subjects with OA grade 2 or 3 features were older and were found to have higher BMI compared with the group with radiological grade 0 (Table 1). 

### 3.2. Genotype Distribution of Investigated SNP

**Figure 1:**
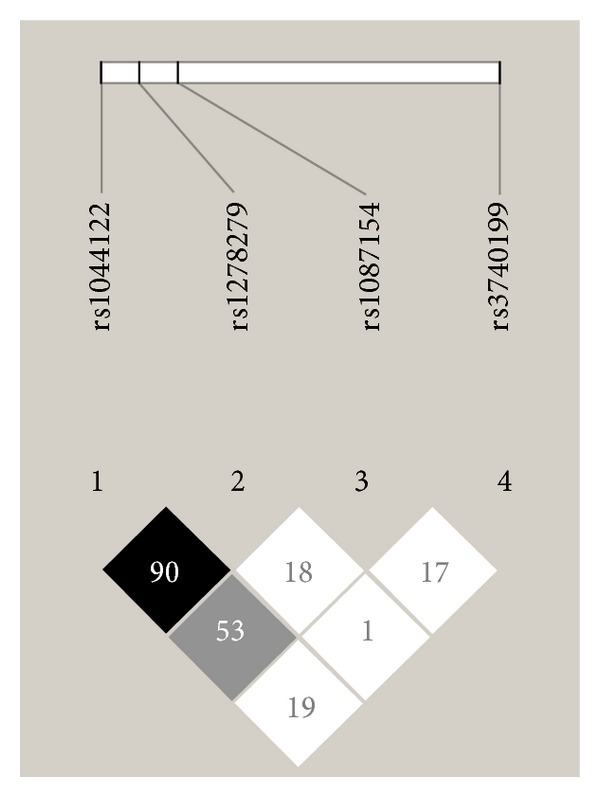
The linkage disequilibrium (LD) across the *ADAM12* gene. The results of LD mapping are generated using Haploview software. The values for D′ between each SNP are presented in each box. Black boxes denote strong LD (D′ > 80%), grey boxes denote weak LD (D′ 50–70%), and white boxes denote very weak or negligible LD (D′ < 50%).

As expected, the distribution of the genotypes of SNPs of *ADAM12* gene conformed to the HWE and the genotyping success rate was 100%. The observed minor allele frequencies (MAF) were 35% for C allele of rs3740199, 50% for C allele of rs1871054, 16% for A allele of rs1278279, and 36% for G allele of rs1044122. Our finding suggests that the distribution of allele frequencies of genotyped SNP in Estonian population is similar to those reported by other studies of European descent (*P* = nonsignificant). Interestingly, the degree of linkage disequilibrium (LD) within *ADAM12* gene was found to be predominantly weak (D′ 0.01–0.53) strong LD (D′ > 0.8) across was found only between rs1044122 and rs1278279 showing that these two polymorphisms belong to one haploblock (Figure 1). 

### 3.3. Association of ADAM12 Polymorphisms with Different Grades of Radiological KOA 

#### 3.3.1. Overall rKOA Survey (Grade 0 versus Grades 1–3)

When the genetic analysis was performed by comparing subjects without rKOA features to those with grades 1–3, only rs1044122 carried the risk for rKOA. The A allele of rs1044122 was associated with radiographic OA in TFJ (OR   1.56, 95%CI 1.09–2.23, *P* = 0.016, and corrected P* = 0.033) in females, predominantly owing to the presence of osteophytes (OR  1.52, 95%CI 1.06–2.16, *P* = 0.022, and corrected P* = 0.046). 

For other SNPs, no statistically significant associations with rKOA were found.

#### 3.3.2. Early rKOA Survey (Grade 0 versus Grade 1)

To investigate more carefully the genetic risk of early OA we carried out separate analysis for subjects with grade 1 of OA. It turned out that the major (ancestral) allele A of rs1044122 was associated with increased risk of early OA occurrence in TFJ, in female patients predominantly (OR 1.63, 95%CI 1.12–2.37, Table 2). Separate analysis of two main radiographic features of KOA reveals that the genetic risk was associated with the presence of osteophytes in TFJ (OR 1.57, 95%CI 1.08–2.29, Table 2), whereas the greatest risk was noticed for AA genotype (OR 3.81, 95%CI 1.42–10.23, Table 2). 

#### 3.3.3. Advanced Radiological KOA (Grade 0 versus Grade ≥ 2)

The C allele of rs1871054 carried the higher risk for the development of advanced (grade ≥ 2) radiological OA only in male subjects. In our study group, this allele was associated with global score of KOA (OR 2.82, 95% CI 1.2–6.67, Table 3) mostly because of radiological changes in TFJ (OR 3.82, 95%CI 1.36–10.73, Table 3). Again, from radiographic rKOA features, the genetic risk was associated with osteophytosis in TFJ (OR 3.03, 95%CI 1.11–7.53, Table 3).

The study power for investigated SNPs varied from 88–91% at minimum OR 1.3.

### 3.4. Association of ADAM12 Haplotypes with rKOA

Haplotype analysis revealed that two haplotypes associated with the increased risk of rKOA (Table 4). Haplotype CCAA (rs3740199, rs1871054, rs1278279, and rs1044122) in male subjects was found to be related to increased risk of OA in TFJ (*P* = 0.014, Table 4). From two main rKOA features risk was predominantly associated to the occurrence of osteophytes (*P* = 0.014, Table 4). The second haplotype GCGG—most frequently calculated—was associated with JSN (loss of cartage matrix) in women (*P* = 0.048, Table 4).

## 4. Discussion

The genetic background is important determinants of OA. An extensive functional genomic research (DNA and RNA) on relevant joint tissue, cell, and animal models is needed to discover novel unknown members and elucidate mechanisms of current OA susceptibility genes and pathways [19]. Identification of OA susceptibility genes could make it possible in the future to predict disease phenotypes as well to construct OA prediction models based on genotype information [20].

The association of ADAM12 polymorphisms with OA has sparsely been investigated. Four SNPs in *ADAM12*—rs3740199, rs1871054, rs1278279, and rs1044122—have been investigated in relation to OA so far [6, 7, 17, 21]. Up to now, *ADAM12* was reported to be associated with the increased risk of the development of KOA in females from Chingford study, UK [6]. Valdes et al. found that rs3740199 SNP in *ADAM12* carried the risk for osteophyte development and progression [6]. However, an extensive GOAL study (UK) was later unable to replicate the association [7]. In another British case-control study the association between ADAM12 SNPs and KOA was insignificant but one haplotype (CAAT) in the *ADAM12* gene significantly increased risk of KOA in men as well in women [17]. Therefore, the impact of *ADAM12* on OA pathogenesis has remained controversial and the possible association should be investigated in more detail and in different populations. 

This study was designed to investigate the possible contribution of selected genetic variants of the *ADAM12* gene to rKOA in a middle aged population cohort, where early rKOA is expected to be predominant. Moreover, evaluation of the *ADAM12* genetic contribution to different pathophysiological processes of OA (osteophyte formation, joint space narrowing) was one of the main tasks of our study.

Notably, in the current study two *ADAM12* SNPs were related to increased osteophyte risk in both early and late rKOA. First of all, the rs1871054 was associated with the appearance of osteophytes of advanced grade only in males. Unlike the late OA genetic susceptibility, early OA changes (osteophytes of grade 1) were found predominantly in woman and related to synonymous polymorphism rs1044122 (p.Ala824Ala). The relation of *ADAM12 *genetic variants to osteophytosis observed in the current study is supported by our previous research, demonstrating association between *ADAM12 *and osteophytes on gene [22] and protein level [10].

The osteophyte formation represents repair attempts and seems to be primarily a process of neochondrogenesis of mesenchymal stem cells present in the periosteum. Also of cells derived from synovial lining and intramembranous bone formation can contribute to the definitive osteophyte [23]. Meltrin alpha (ADAM12) protein was shown to induce osteoclast formation [24], and this could be a possible link to bone remodelling.

The discordance between our results and other studies on *ADAM12* could be explained by differences in the study design and by the investigated cohorts. In GOAL study, the cases were defined as joint JSN ≥ 2 and/or osteophyte score ≥1. Their controls were recruited from a list of patients with suspected urological problems without evidence of radiological KOA. By contrast, we compared the KOA grade 0 subjects with those of grades 1–3 from the same population cohort. We support the view that summarization of the JSN and osteophytes into the global OA grade may dilute/veil the information on early OA [25]. Therefore we used the Nagaosa et al. system where rKOA features are evaluated in the three knee compartments providing a better assessment of early OA and its progression [12].

In general, the Estonian population can be considered as an open population. Here, it should be noted that selecting a Southern Estonia cohort is the strength of the current study as the population of this region has had a stable residence for two centuries. Thus, we investigated a small but homogenous cohort. 

It is well known that the size of the sample may have an impact on the results. In the current study, we failed to detect an association between rs3740199 in *ADAM12* and rKOA was reported previously by us in initial significantly smaller group [20]. The reason for this discrepancy probably could be related to the putative controversial contribution of this variant to the KOA in men and women. For example, in our study, we found that haplotype containing C allele of rs3740199 carried the risk of OA in men, and haplotype containing G allele increased the risk in women. Also, it has been hypothesized that this SNP acts as a modulator of genetic susceptibility only in the presence of other alleles [16].

The LD analysis of *ADAM12* SNP in our material demonstrated that two of them (rs1278279 and rs1044122) belong to the same haploblock. Rather weak LD among the other SNP indicates that in genetic risk assessment rs1871054, rs3740199, and one of variants belonging to haploblock must be evaluated separately.

Up to now we have to admit that most individual genetic associations are relatively modest. However, additive information from a number of genetic variants associated with knee OA could result in substantially greater risk of OA. It is known from earlier studies and confirmed by our data that the genetic effect might be gender dependent. Thus, the new models to predict inheritance patterns of KOA have to include effect of many genes and other parameters.

There are limitations to the current study. A total of 438 subjects may seem a relatively small number for detecting weak genetic associations. Indeed, single population-based studies cannot often provide a sufficient number of participants of certain age. However, the population-based design is the main of advantage in this case. As it is known, in large genomewide linkage studies as well as in smaller case-control studies it is quite difficult to ensure homogeneity of radiographic/clinical diagnosis (e.g., heterogeneity in imaging technique, insufficient radiographic evaluation of controls, and prone to selection and recall bias). Our study provides the data of precise evaluation and identical clinical/diagnostic testing of all recruited participants. Additionally, recruitment of middle aged subjects and focusing on early rKOA could potentially reduce the confounding comorbidities in older cohorts. 

In conclusion, our data support the consideration of *ADAM12* gene as potentially associated with increased risk of KOA during the early and late stages of diseases and that is seems to be predominantly related to osteophytosis (bone remodelling and neochondrogenesis). We believe that further investigation of *ADAM12* in other populations is needed, and performing meta-analysis of published data in the future should provide a better understanding of potential of the this gene contributing to OA risk. 

## Figures and Tables

**Table 1:** The summary of radiographic knee OA evaluation and characteristics of study group.

	OA in TFJ			OA in PFJ			OPH		
	Grade 0 (*N* = 211)	Grade 1 (*N* = 188)	Grade 2-3 (*N* = 39)	Grade 0 (*N* = 226)	Grade 1 (*N* = 177)	Grade 2-3 (*N* = 35)	Grade 0 (*N* = 185)	Grade 1 (*N* = 205)	Grade 2-3 (*N* = 48)
Females, %	68	71	67	68	73	62	67	73	63
Age Mean (±SD)	44.3 (5.9)	46.1 (5.5)	48.2 (5.5)*	44.3 (6.0)	46.5 (5.3)	47.6 (5.4)*	44.4 (5.9)	4.9 (5.5)	47.9 (5.6)*
BMI Mean (±SD)	25.9 (4.6)	28.6 (4.8)	30.1 (5.7)*	26.3 (4.6)	28.2 (5.2)	29.3 (5.5)*	25.6 (4.5)	28.2 (4.5)	29.8 (5.3)*

OA in TFJ: radiographic osteoarthritis in tibiofemoral joint; OA in PFJ: radiographic osteoarthritis in patellofemoral joint; OPH: osteophytes; BMI: body mass index; *P value < 0.001 (compared to the group with radiographic stage 0).

**Table 2:** Statistically significant associations in group with early knee OA (grade = 1) with *ADAM12 *gene SNPs (were observed only for rs1044122).

	Grade 0	Grade 1	OR (95% CI)	*P* value	Corrected *P* value**
Rs1044122, whole group	OA in TFJ				

A allele	61%	68%	1.37 (1.01–1.87)	0.042	0.087
AA	82 (39%)	79 (43%)	2.43 (1.17–5.05)	**0.017**	**0.036**
AG	93 (45%)	92 (50%)	3.26 (1.10–4.62)	0.026	0.054
GG	34 (16%)	14 (7%)	1*	1*	

Rs1044122, females	OA in TFJ				

A allele	60%	70%	1.63 (1.12–2.37)	**0.018**	**0.038**
AA	53 (37%)	58 (44%)	3.78 (1.5–9.54)	**0.005**	**0.010**
AG	66 (46%)	65 (50%)	2.92 (1.18–7.23)	**0.021**	**0.043**
GG	24 (17%)	8 (6%)	1*	1*	

Rs1044122, females	OPH in TFJ				

A allele	60%	70%	1.57 (1.08–2.29)	**0.018**	**0.038**
AA	61 (37%)	50 (45%)	3.81 (1.42–10.23)	**0.008**	**0.017**
AG	76 (47%)	56 (50%)	3.09 (1.17–8.16)	**0.023**	**0.048**
GG	26 (16%)	6 (5%)	1*	1*	

OA in TFJ: radiographic osteoarthritis in tibiofemoral joint; OPH in TFJ: osteophytes in tibiofemoral joint; *GG genotypes were analysed as base; **for multiple comparison correction Benjamini and Yekutieli method was used; in bold are shown statistically significant *P* values.

**Table 3:** Statistically significant association of rs1871054 in *ADAM12* gene with advanced (grade ≥ 2) knee radio features (were found only on males).

	Grade 0	Grade ≥ 2	OR (95% CI)	*P* value	Corrected *P* value**
rs1871054, males	OA				

C allele	44%	68%	2.82 (1.20–6.67)	**0.018**	**0.038**
CC	8 (16%)	10 (50%)	7.86 (1.30–47.69)	0.025	0.052
CT	29 (57%)	7 (35%)	1.39 (0.28–7.02)	NS	
TT	14 (27%)	3 (15%)	1*	1*	

rs1871054, males	OPH				

C allele	45%	70%	2.67 (1.11–6.44)	**0.029**	0.06
CC	11 (18%)	8 (61%)	5.31 (0.94–29.87)	NS	
CT	33 (55%)	5 (38%)	1.01 (0.19–5.48)	NS	
TT	16 (27%)	3 (23%)	1*	1*	

rs1871054, males	OA in TFJ				

C allele	48%	73%	3.82 (1.36–10.73)	**0.011**	**0.023**
CC	12 (18%)	8 (62%)	10.11 (1.29–79.45)	0.028	0.058
CT	35 (53%)	3 (23%)	1.26 (0.17–9.54)	NS	
TT	19 (29%)	2 (15%)	1*	1*	

rs1871054, males	OPH in TFJ				

C allele	47%	73 %	3.03 (1.11–7.53)	**0.018**	**0.038**
CC	15 (20%)	8 (53%)	6.63 (1.15–38.17)	0.034	0.07
CT	39 (53%)	5 (323%)	1.66 (0.30–9.40)	NS	
TT	20 (27%)	2 (15%)	1*	1*	

OA: global grade of radiographic knee OA in tibiofemoral joint and/or patellofemoral joint; TFJ: tibiofemoral joint; OPH: osteophytes; *TT genotypes were analysed as base; **for multiple comparison correction Benjamini and Yekutieli method was used; in bold are shown statistically significant *P* values.

**Table 4:** The genetic association of *ADAM12* haplotype (rs3740199, rs1871054, rs1272278, and rs1044122) and radiographic knee OA features.

Haplotype	Features	Grade 0^†^	Grade ≥ 2^††^	*P* value	sim *P* value*
CCAA, whole group	OA in TFJ	1.4%	5.6%	0.005	0.024
	OPH	1.4%	3.8%	0.018	0.040
	OPH in TFJ	1.4%	5.5%	0.004	0.030

CCAA, males	OA in TFJ	1.9%	15%	0.0001	0.014
	OPH	1.3%	11%	0.005	0.028
	OPH inTFJ	1.2%	15%	0.0001	0.014

GCGG, females	JSN	19%	43%	0.040	0.048

OA: radiographic osteoarthritis in tibiofemoral joint (TFJ); OPH: osteophytes; JSN: joint space narrowing; ^†^calculated haplotype frequencies in subjects with no radiographic changes (grade 0); ^††^calculated haplotype frequencies in subjects with late radiographic knee OA features; *by 10 000 simulations.
